# Resilience of Loin Meat Microbiota and of Resistance Genes to a Chlortetracycline Treatment in Weaned Piglets

**DOI:** 10.3390/antibiotics13100997

**Published:** 2024-10-21

**Authors:** Xavier C. Monger, Linda Saucier, Alex-An Gilbert, Sophie Gosselin, Éric Pouliot, Sylvain Fournaise, Antony T. Vincent

**Affiliations:** 1 Département des Sciences Animales, Faculté des Sciences de l’Agriculture et de l’Alimentation, Université Laval, Québec, QC G1V 0A6, Canada; xavier.monger.1@ulaval.ca (X.C.M.); linda.saucier@fsaa.ulaval.ca (L.S.); alex-an.gilbert.1@ulaval.ca (A.-A.G.); sophie.gosselin.8@ulaval.ca (S.G.); 2 Institut sur la Nutrition et les Aliments Fonctionnels, Université Laval, Québec, QC G1V 0A6, Canada; 3 Centre de Recherche en Infectiologie Porcine et Avicole, Faculté de Médecine Vétérinaire, Université de Montréal, St-Hyacinthe, QC J2S 2M2, Canada; 4 Institut de Biologie Intégrative et des Systèmes, Université Laval, Québec, QC G1V 0A6, Canada; 5 Olymel S.E.C., Boucherville, QC J4B 5Y1, Canada; ericpouliot@olymel.com (É.P.); sylvainfournaise@olymel.com (S.F.)

**Keywords:** antibiotic resistance, food microbiology, meat, metagenomics, swine

## Abstract

Objectives: This project studied the impact of a chlortetracycline treatment in weaning piglets on the taxonomy and antibiotic resistance gene (ARG) content of the microbiomes on carcasses and loins. Methods: Two groups of piglets from two farrowing barns with either an average or a lower sanitary health status were used. Each group was divided in half: a control group and a treatment group receiving feed supplemented with 660 g of chlortetracycline per tonne for 21 days. The piglets then went through fattening and were sent to the abattoir when they reached the targeted slaughter weight. Results: The microbiomes of the pig carcasses and loins were sampled, and DNA was extracted and sequenced with a whole-genome approach. The microbiomes of the carcasses differed depending on the farrowing barn source in both taxonomical composition and ARG content; however, the microbiomes on the loins were similar, regardless of the farrowing barn source and the treatment group. Conclusions: While there were differences in the carcass microbiomes between treatments after processing by the abattoir, the loin microbiomes were consistent and unaffected by treatment with chlortetracycline or the sanitary status of the farrowing barn.

## 1. Introduction

For many years, antibiotic resistance has been a priority of national health organisations [1]. Antibiotic resistance is a naturally occurring phenomenon in the bacterial population [2], and organisms that produce antibiotics need to be resistant, otherwise they would create an environment that is harmful to themselves. However, ongoing antibiotic use for human health and agricultural purposes has contributed to the selection of bacteria carrying antibiotic resistance genes (ARGs) and increased their abundance [3,4]. Because the efficacy of antibiotic treatments is being threatened by resistant bacteria, there is a growing will to reduce antibiotic use to mitigate the selection of resistance.

The magnitude of agricultural activity means that agriculture has a high use of antibiotics, along with the medical field [5,6]. Considerable work has been carried out to reduce antibiotic administration at the farm level and, notably, antibiotics as growth-promoting factors have been banned in many countries [6]. The One Health approach—which states that the environment, animals, and humans are interconnected [7]—to antibiotic resistance has promoted a more global and sustainable process to reduce resistance development; therefore, the impacts of antibiotic interventions in agriculture and human medicine are increasingly studied [8]. The growing concern that ARGs could be transferred to humans through animal products (e.g., manure used for fertilisation or meat) has fostered research on how to mitigate antibiotic resistance in a wide variety of environments [9].

Pig production is one of the most prevalent animal-rearing activities worldwide, and the swine industry is a major consumer of antibiotics [5]. Therefore, this industry has been challenged to reduce its antibiotic usage without compromising the health and welfare of the animals. During critical periods in their growth, pigs are particularly susceptible to infection. At weaning, it is still common to administer antibiotic treatment, as stress on the piglet gut microbiome increases their susceptibility to colonisation by pathogens, often resulting in signs of sickness and discomfort including diarrhoea [10,11,12]. Consequently, not treating pigs with antibiotics can lead to animal welfare issues, high mortality rates, and economic losses for producers [13].

The weaning period takes place close to the beginning of the piglet’s lifespan (starting 21 days after birth), which is months before slaughter (151–174 days after birth). Some studies have demonstrated that gut microbiomes can, at least partially, revert towards a pretreatment state of reduced ARG content over time, for both pigs and humans, although no precise duration of recuperation has been established [14,15]. Moreover, Laforge et al. demonstrated that a small proportion of bacteria present on meat originates from the farm [16]. However, there is limited knowledge regarding antibiotic resistance resilience in the pig microbiome. 

This study investigates whether antibiotic resistance that develops in treated piglets at weaning persists along the value chain if no other antibiotic treatments are administered, and if this antibiotic resistance is ultimately transferred onto the meat after slaughter. More precisely, we studied the impact of chlortetracycline treatment at weaning in piglets on the taxonomy and ARG content of microbiomes on carcasses and loins. Two farrowing barns with different sanitary health statuses were selected by clinical veterinarians based on their health inspection history. For each farrowing barn, we followed a group of piglets treated with chlortetracycline at weaning and a control group that was not treated. The loins of the pigs were collected at the abattoir and the bacterial community on their surfaces was sampled. DNA sequencing of the extracted DNA was used to determine how the presence of ARGs on the meat changed over time.

## 2. Results

### 2.1. Taxonomical Composition

**Figure 1:**
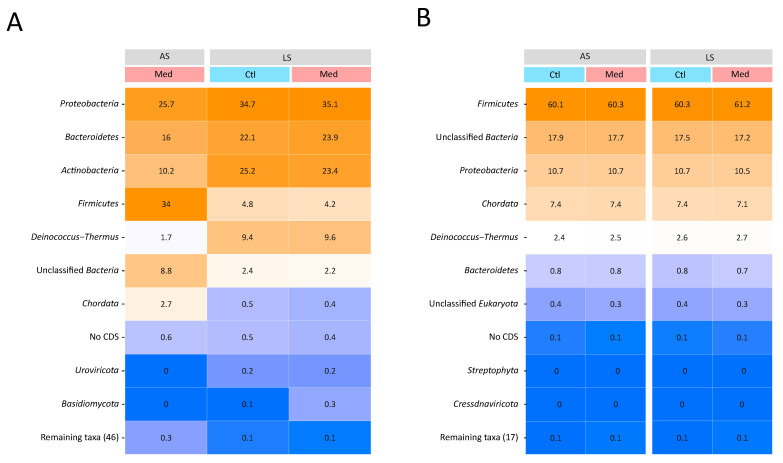
Taxonomic composition of the microbiomes on (**A**) carcass and (**B**) loin samples according to the farrowing barn source and treatment. Samples were grouped by the farrowing barn source and its respective sanitary health status: average (AS) or lower (LS). The samples from each farrowing barn were also grouped by the treatment administered to the piglets: control (Ctl) or medicated with chlortetracycline at weaning (Med). Colour gradients represent the relative abundance of each taxon and range from blue (0%) to orange (100%). The most abundant phyla are displayed on the *y* axis.

The taxonomic profiles of the samples taken from the Med carcasses differed depending on the farrowing barn source (Figure 1). Unfortunately, the carcass sample for the control group of the AS farrowing barn did not yield enough DNA to be sequenced and, therefore, was excluded from the analyses. Because very few reads originated from microbial DNA (Supplementary Table S2), many taxa could not be assigned beyond the phylum level; therefore, taxonomic analyses focused mainly on phyla. The samples taken from the loins, regardless of the farrowing barn source or treatment, had a nearly identical taxonomic composition and were largely dominated by the *Firmicutes* phylum. This phylum was also highly abundant on the carcasses from the AS farrowing barn, but not on carcasses from the LS farrowing barn, where *Proteobacteria* was the prevailing phylum (Figure 1). The microbial communities on the carcasses also had different microbial composition and abundance from those on the loins. The microbiomes of the carcasses were more evenly distributed across multiple taxa, while 60% of the sequences from the loin microbiomes belonged to only one phylum (*Firmicutes*). The *Bacteroidetes* and *Actinobacteria* phyla both constituted more than 10% of the microbial community of the carcasses, while the *Bacteroidetes* phylum accounted for less than 1% of the microbial community of the loins, and the *Actinobacteria* phylum was not among the ten most abundant phyla. The microbiomes of the Ctl and Med carcass samples from the LS farrowing barn were similar in composition, while the microbiome of the Med carcass sample from the AS farrowing barn had a lower proportion of *Proteobacteria* (25.7% AS-Med; 34.7% LS-Ctl, 35.1% LS-Med) and *Actinobacteria* (10.2% AS-Med; 25.2% LS-Ctl, 23.4% LS-Med) and a higher proportion of *Firmicutes* (34% AS-Med; 4.8% LS-Ctl, 4.2% LS-Med).

**Figure 2:**
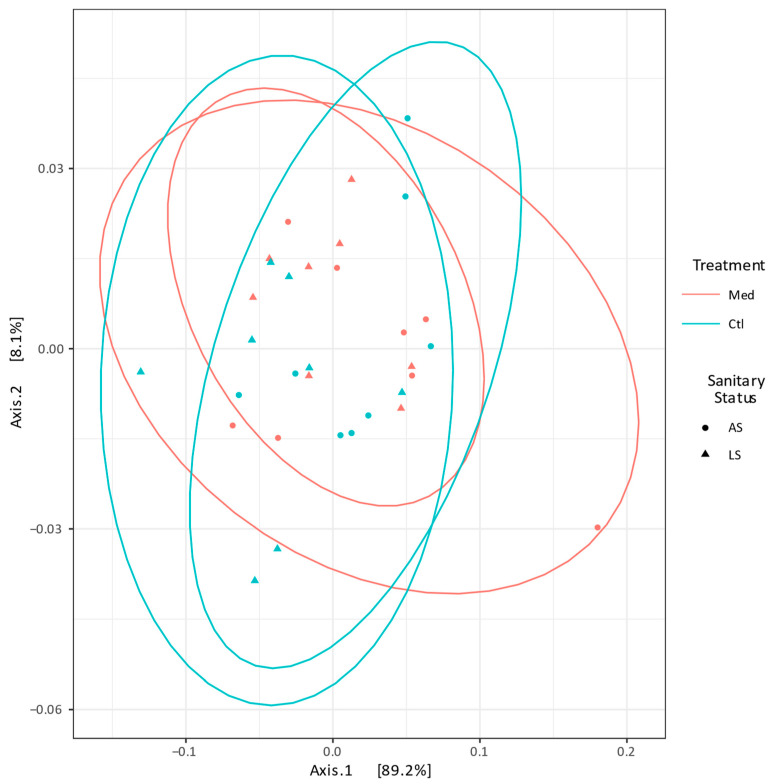
Principal coordinate analysis plot of the β-diversity (Bray–Curtis dissimilarity) of the microbial communities of the loin samples. Samples were grouped by the farrowing barn source and its respective sanitary health status: average (AS) or lower (LS). The samples from each farrowing barn source were also grouped by the treatment administered to the pigs: control (Ctl) or medicated with chlortetracycline at weaning (Med). Each point represents a sample. The distance between points reflects the difference in microbial composition between samples; closer points indicate higher similarity. The principal axes represent dimensions that maximise variance among samples. Ellipses represent 95% confidence intervals.

Alpha diversity was higher on the carcasses (Shannon index > 2 for all samples) than on the loins (Shannon index < 1.40 for all samples). However, no significant difference in α-diversity was observed in the loins for either treatment (*p* = 0.2 for the LS farrowing barn and *p* = 0.24 for the AS farrowing barn) from either farrowing barn source (Supplementary Figure S1). A principal coordinate analysis of the β-diversity shows that the composition of the loin microbiota was similar in the loins from different farrowing barn sources and treatments. No significant difference between the microbial composition of the treatment groups was observed for loins from the AS (*p* = 0.46) or the LS (*p* = 0.51) farrowing barn sources (Figure 2).

### 2.2. Antibiotic Resistance

**Figure 3:**
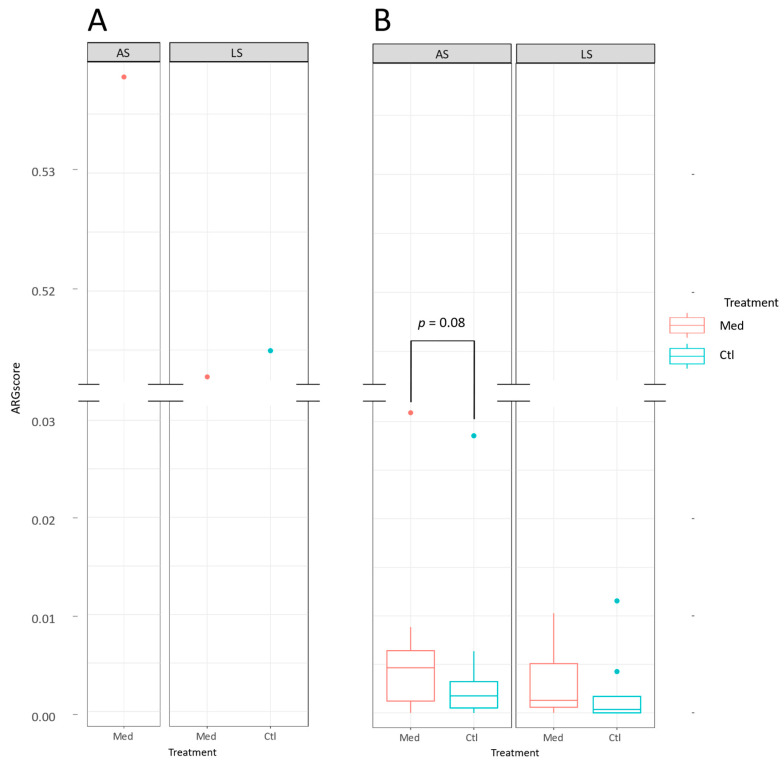
Antibiotic resistance genes scores (ARG scores) of (**A**) carcass and (**B**) loin samples. Samples were grouped by the farrowing barn source and its respective sanitary health status: average (AS) or lower (LS). The samples of each farrowing barn source were also grouped by the treatment administered to the pigs: control (Ctl) or medicated with chlortetracycline at weaning (Med). The median is indicated by the bar in the box. The box extends from the 1st to the 3rd quartiles, while the whiskers extend to the maximum and minimum values. An interquartile range of 1.5 was used to determine outliers, which are represented by dots.

Antibiotic resistance gene scores (ARG scores) were calculated for each sample. This score was normalised by the number of reads in each sample (Figure 3). There was a considerable difference between the ARG scores of the carcasses and the loins. The microbiomes of the carcasses had many more ARGs (ARG score > 0.51 for all samples) than the microbiomes of the loins (ARG score < 0.035 for all samples). There was a tendency for the ARG scores to be higher on the loins of the Med treatment than on the loins of the Ctl treatment for the AS farrowing barn (*p* = 0.08). No significant difference was observed between the ARG scores of loin samples from different treatments (Med, Ctl) from the LS farrowing barn (*p* = 0.64). Additionally, when each treatment was compared between the two farrowing barn sources, there was no significant difference in ARG scores for either one (*p* = 0.58 for the Med treatment and *p* = 0.33 for the Ctl group). In the view of this lack of difference, loins from each treatment for both piglet sources were combined and the ARG scores did not significantly differ between treatments (*p* = 0.3), suggesting that in the end, the ARG scores on the loins were not influenced by the antibiotic treatment given at weaning.

**Figure 4:**
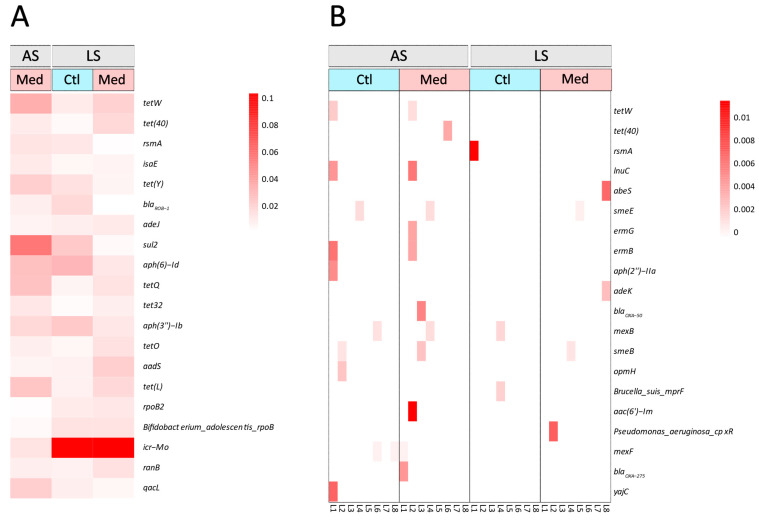
Heatmap based on the score of antibiotic resistance genes (ARG score) of the 20 most prevalent ARGs in (**A**) carcass and (**B**) loin samples. Samples were grouped by the farrowing barn source and its respective sanitary health status: average (AS) or lower (LS). The samples from each farrowing barn were also grouped by the treatment administered to the piglets: control (Ctl) or medicated with chlortetracycline at weaning (Med). For the loins, replicates of all eight individual pooled samples for each subgroup are displayed on the *x* axis, ranging from L1 to L8. The most prevalent resistance genes are displayed on the *y* axis. Colour gradient is based on the ARG score of each ARG in each sample and ranges from white (0) to dark red (0.012).

The profile of ARGs was different and richer for the carcasses compared with that of the loins (Figure 4). Only three genes were among the 20 most abundant ARGs in both sample types: two tetracycline resistance genes (*tet*(w) and *tet*(40)) and one gene conferring resistance to diaminopyrimidine, phenicol, and fluoroquinolone (*rsmA*). While the most abundant ARGs in the carcass samples were detected in all samples (Figure 4A), none of the most abundant genes in the loins were detected in all samples (Figure 4B). The genes that were detected in the highest number of loin samples were *smeB* and *mexB*, which code for proteins that are part of multidrug efflux pump complexes; they were detected in three samples out of eight. The most abundant gene detected in the carcasses from the LS farrowing barn group (*icr-Mo*, a colistin resistance gene) was not detected in the microbiome of the loins obtained from the same carcasses. Similarly, the most abundant gene detected on the AS farrowing barn carcasses (*sul2*, a sulfonamide resistance gene) was also not observed in the microbiome of the loins from the same carcasses. The gene *icr-Mo* was present at a higher abundance on the carcasses from the LS farrowing barn (ARG score > 0.1) than on the carcasses from the AS farrowing barn (ARG score = 0.013). Among the loin microbiomes, no clear difference in ARG composition can be established between the treatments or farrowing barn sources.

## 3. Discussion

This study aimed to establish the impact of a chlortetracycline treatment for piglets at weaning on the microbiome and ARG content of their carcasses and loins after processing at the abattoir. We used animals that originated from two farrowing barns having different sanitary health statuses. This study provides valuable information on how the microbiomes from those animals changed during processing from carcasses to loins.

The microbiomes of the carcass samples from both groups originating from the LS farrowing barn had a similar taxonomic composition and ARG score. The microbiome from the Med carcass sample from the AS farrowing barn had a different taxonomic composition and a higher ARG score than the Med carcass samples from the LS farrowing barn. This suggests that the farrowing barn of origin may influence the microbiome of the carcasses as well as the ARG content. An episode of coughing and diarrhoea caused by influenza was observed in both experimental groups at the nursery for animals coming from the LS farrowing barn, but no treatment was required and the animals recovered. Conversely, pigs from the AS farrowing barn were prescribed a supplement of vitamins and selenium, salicylic acid, and iodine by the veterinarian for a coughing episode (Table 1). These events might have influenced the variations observed on the carcasses between the two farrowing barns. In an abattoir in the province of Québec with similar practices, the microbiomes of the carcasses did not differ with farm of origin [17]. However, in that study, all samples were taken on the same day, and the microbiota was analysed with 16S rRNA gene sequencing metabarcoding. Another study conducted in an abattoir in the United States demonstrated that the slaughter group was responsible for a very small portion of the differences in the resistome of carcasses after processing at the abattoir. However, in that study, pigs from all groups were slaughtered on the same day, which could in part explain why the carcass microbiomes were similar after processing [18]. In experiments that have a different sampling day for each group, the effect of sampling day cannot easily be distinguished from the effect of farm of origin. Inversely, in experiments designed with a single sampling day, if the effect of the sampling day is greater, it could mask the effect of the farm of origin. However, it is also possible that cross-contamination between slaughter groups occurs when all slaughter groups are processed on the same day. Therefore, a study with multiple farms, including ones with a higher sanitary status, and multiple sampling days for each treatment group from each farrowing barn source is required to confirm the effect of sampling days and farrowing barn of origin.

Laforge et al. previously demonstrated that only a small portion of bacteria found on meat can have its origin traced back to the farm [16]. The very similar microbial community on the loins confirms this, and also suggests that the microbial communities on loins from this abattoir are fairly consistent regardless of the farrowing barn of origin. The difference in microbial communities found on the carcasses originating from different maternities suggests that the similarity in the loin microbiota is not a result of a similar farrowing barn source. Because the microbial communities of loins were similar between farrowing barn sources, the similarity between treatments (Ctl and Med) could be a result, at least in part, of the replacement of farm microbiota on loins with the persistent microbiota from the abattoir. The microbial phyla observed on meat samples have been reported in varying proportions on pork loins [19,20]. Notably, the relative abundance of microbial species might be affected by the low number of reads in the samples after the removal of pig genomic DNA sequences (Supplementary Table S2). However, the taxonomic composition of the microbiota tends to be relatively stable with varying sequencing depth [21]. Also, our analysis focused mainly on comparing samples of equal sequencing depth, not on absolute composition. Therefore, the low number of reads does not change the interpretation of the results.

The high difference in the ARG content of the carcasses compared with the loins suggests that most of the ARGs present on the carcasses were not transferred to the loins. The chlortetracycline treatment did not seem to have a marked impact on the ARG content of the loins. However, there was a slight trend towards higher ARG scores in the loins of the Med group compared with the Ctl group from the AS farrowing barn. The differences in ARG profiles between the carcasses and the loins suggests that they might not be a result of the farrowing barn source or fattening farm microbiome, and also that the microbiome of the carcass is not transferred to the meat after processing. To verify whether the sanitary health status influenced the impact of the treatment of piglets on the loin microbiome, more studies are needed with a higher number of replicates and testing farrowing barns with a high health status.

We observed tetracycline resistance genes in this study; however, they were not more abundant than genes conferring resistance to other classes of antibiotics, even though the antibiotic used in this experiment belongs to the tetracycline class. This suggests that the resistome of the loin microbiota was not greatly influenced by the selection pressure caused by the treatment. Moreover, multiple studies have established that tetracycline resistance genes are found widely across samples from pig production [22,23,24]. The lack of impact of antibiotic treatment on the loin microbiomes might be explained by the constant microbiota acquired from the abattoir. In contrast, the lack of difference between the taxonomical composition of the microbiomes and the resistomes of the carcasses from chlortetracycline-treated animals compared with the control group for the LS farrowing barn group could be explained by the long period between treatment and slaughter (at least 95 days for all groups; Supplementary Table S1); this could have allowed the microbiota to recover during the fattening phase when no antibiotic was used. Although this study did not determine a definitive time required for the microbiome to return to a pretreatment state, some studies suggest that it may be weeks to a few months [14,15,25,26]. To confirm the effect of microbiome recovery, a similar study of loin microbiota that also monitors the ARGs in the faeces of the pigs would be necessary. More studies are also needed to establish whether this effect is also observed with antibiotics other than chlortetracycline. Monger et al. reported that in the pig/pork value chain, antibiotic resistance decreases in pig meat as the product moves further away from the site and time of antibiotic treatment [15].

In conclusion, the results from this study confirm those from Laforge et al. that a large portion of the pig microbiota can be replaced with the abattoir microbiota during the processing of the meat [16]. This confirms the importance of food safety and microbial control at the plant level and the efficacy of their preventive control plan (HACCP plan). Although the carcass microbiota was different between pigs from the Med group of the AS farrowing barn and pigs from the LS farrowing barn, the loin microbiota was constant across the different groups of pigs. Treatment with chlortetracycline at weaning did not impact the taxonomical composition nor the ARG content of the loin microbiomes. In this study, the sanitary health status of the farrowing barn did not affect the microbiomes of pork loins. However, a study with more farrowing barn sources, including those with a high health status, is necessary to confirm this lack of effect. Furthermore, a next challenge would also be to identify where the antibiotic resistance cut-off points occur in the pork value chain.

## 4. Materials and Methods

### 4.1. Animals and Treatment

All experimental procedures involving live animals were approved by Université Laval’s Animal Use and Care Committee (2019-310/VRR-19-036), which strictly adheres to the Guidelines of the Canadian Council on Animal Care [27].

Piglets were of the same genetics (offspring of Yorkshire × Landrace sows sired with Duroc boars) and were followed from nursery to slaughter; herd management information is presented in Table 1. The same feeding programme was followed throughout the experiments (Supplementary Table S1). All piglets were injected intramuscularly into the neck with 12 mg of trimethoprim and 60 mg of sulfadoxine per piglet in a final volume of 0.3 mL (Borgal, Merck Animal Health, Madison, NJ, USA) within the first 15 h after birth, and were also injected during growth with 2 mL of a combined vaccine. The first vaccine was a combination of porcine circovirus (PCV) type 2b vaccine and *Mycoplasma hyopneumoniae* bacterin (Circo/MycoGard^®^; Pharmgate Animal Health LLC, Wilmington, NC, USA). The second vaccine contained *Lawsonia intracellularis* bacterin (Porcilis^TM^ ileitis; Merck Animal Health, Madison, NJ, USA).

This study was part of a larger microbiological experiment aiming at characterising the microbial ecology of the whole pork value chain; the experiments were conducted in two phases. All animals were processed within a 16-month period; animals from the commercial farrowing barn with an average health status (AS) were processed from July to December 2020, and animals from the commercial farrowing barn with a lower health status (LS) were processed from June to October 2021. The two farrowing barns were selected from 126 farms by experienced veterinarians based on their health history. The growth period was conducted in a single commercial experimental barn, and the fattening phase was conducted in different commercial experimental barns because of restricted availability imposed by the COVID-19 pandemic. Piglets of each farrowing barn source were divided into two experimental groups, a group medicated with antibiotics at weaning (Med) and a control group (Ctl) which did not receive the treatment. The medicated group from each farrowing barn source (Med) received a prophylactic administration of 660 g of chlortetracycline calcium complex per tonne of feed (Deracin^®^ 22% Granular Premix; Pharmgate Animal Health LLC, Wilmington, NC, USA) at weaning; the feed containing the antibiotic was provided ad libitum for a period of 21 days. The control group (Ctl) from each farrowing barn source did not receive the chlortetracycline treatment in its feed.

Weaned piglets were allocated according to average weight into pens located in two identical and adjacent rooms (15 piglets per pen; 0.29 m^2^/piglet). The two groups were housed in different rooms to avoid microbial cross-contamination. At the end of the growth phase, pigs were transported to a fattening barn. Experimental groups were transported separately in two trips to avoid microbial cross-contamination, with the Ctl group transported first.

At the finishing farm, AS farrowing barn pigs were distributed into 40 pens located in two separate but similar rooms (20 pens/treatment; 20 pens/room; 25 pigs/pen; 0.55 m^2^/pig). LS farrowing barn pigs were distributed into 28 pens (14 pens/treatment; 21 pigs/room; 0.72 m^2^/pig). All pigs were fed ad libitum (Supplementary Table S1) and raised until they reached market weight (130–135 kg).

When pigs reached the targeted slaughter weight, barrows were selected by weight and a feed withdrawal was applied (Table 1) before transport to the same federally inspected slaughterhouse. The two treatment groups were transported on different days (one day per treatment) to avoid microbial cross-contamination between groups. Pigs arrived at the slaughterhouse the day before and were the first to be slaughtered on a clean processing line the next morning according to the current commercial practices. Carcasses were refrigerated overnight and were the first to be cut out the next day.

### 4.2. Sample Collection and Processing

The carcass samples were collected as described by Laforge et al. [16]. Briefly, 25 blast-chilled (90 min) carcasses were randomly selected from each experimental group (Table 1) as they entered the cold room for the overnight cooling process. With a sterile pre-humidified sponge (Whirl-Pak^®^ Speci-Sponge^®^ Environmental Surface Sampling Bags; Nasco, Madison, WI, USA), a total surface of 300 cm^2^ was sampled (100 cm^2^ on the hind leg near the anus, 100 cm^2^ on the belly near the front legs, and 100 cm^2^ on the jowl) using a sterile template (3M cattle template, USDA100, 3M Canada, London, ON, Canada). The sponge was pre-humidified with 10 mL of peptone water (peptone water, phosphate-buffered; Milipore Sigma, Oakville, ON, Canada) and another 10 mL of peptone water was added to the sponge before homogenisation with a Stomacher 400C (Seward Laboratory Systems Inc., London, UK) for 2 min at 230 rpm. Then, 12 mL of peptone water homogenate was centrifuged 20 min at 15,000× *g* at 4 °C. The pellet was resuspended in 2 mL of peptone water and samples were stored at −80 °C until DNA extraction.

Prior to DNA extraction, the 25 carcass samples were grouped into five pools of five carcasses to obtain a sufficient quantity of DNA for sequencing. Total DNA was extracted with the QIAamp BiOstic Bacteremia DNA Kit (QIAGEN, Toronto, ON, Canada). After extracting DNA from the five pooled carcass samples, the five resulting DNA samples were pooled again to form a single equimolar sample. Unfortunately, the carcass sample from the AS farrowing barn control group did not provide enough DNA for sequencing.

For each of the four experimental subgroups, 25 left loins were randomly collected at the abattoir and transported to the laboratory on ice. The surface of the loins was sampled with a sterile sponge as described above, and another 30 mL of peptone water was added to the sponge before homogenisation with a stomacher for 2 min at 230 rpm. A 15 mL of portion of that homogenate was centrifuged, and the pellet was resuspended in 2 mL of peptone water. Samples were stored at −80 °C until DNA extraction. Total DNA was extracted with the QIAamp BiOstic Bacteremia DNA Kit (QIAGEN). DNA samples from 16 loins were randomly selected per experimental group, and eight equimolar pools, each with two loin DNA samples, were created and sequenced.

### 4.3. Sequencing and Data Analysis

Library preparation and sequencing on an Illumina NovaSeq 6000 device was performed by the Genome Québec Centre of Expertise and Services (Montréal, QC, Canada). Libraries were generated using the NEBNext Ultra II DNA Library Prep Kit for Illumina (New England BioLabs, Whitby, ON, Canada) as per the manufacturer’s recommendations. Base calling was performed with RTA v3. The programme bcl2fastq2 v2.20 was used to demultiplex samples and generate fastq reads. The optical duplicates in the reads were removed with the clumpify tool from bbmap version 38.96 [28]. The reads were filtered using version 0.23.1 [29], and the sequences were mapped on the pig genome using bowtie2 version 2.4.4 [30] and samtools version 1.17 [31] to exclude pig genome sequences. The reference genome used was Sscrofa11.1 (RefSeq: GCF_000003025.6). The number of reads at each step is presented in Supplementary Table S2. The whole genome shotgun sequencing dataset was deposited in the NCBI Sequence Read Archive database under the accession ID PRJNA1142024.

The reads were analysed and co-assembled with the SqueezeMeta pipeline version 1.5.1 [32]. The SQMtools R package version 1.6.3 [33] was used to import the data into the R software. The vegan R package version 2.6-4 [34] was used for α-diversity analysis, and the phyloseq [35] package version 1.34.0 was used for β-diversity analysis. The β-diversity was analysed with a permutational analysis of variance (PERMANOVA) with 1000 permutations with the adonis2 function of the vegan R package. ARGs were identified by mapping the reads against the Comprehensive Antibiotic Resistance Database (CARD; Alcock et al. [36]) using MetaProtMiner [37], which also provided a score of antibiotic resistance (ARG score) using the number of reads mapped to each ARG normalised by length of gene and number of reads. For ARG scores, a Kruskal–Wallis test and a post hoc Dunn’s test were used for analysis, and the normality of the data was assessed with a Shapiro–Wilk test. Tests were performed using R version 4.0.5 with the stats package version 4.0.5. A *p*-value threshold of 0.05 was used for significance, and a threshold of 0.1 was used for tendencies.

## Figures and Tables

**Table 1:** Herd management from farrowing to slaughter.

Parameters	AS Farrowing Barn ^a^	LS Farrowing Barn
Initial number of piglets	1020	1200
Initial piglet weight (kg)	6.0	6.5
Piglet weight at end of nursery period (Ctl ^b^; kg)	27.0	29.2
Piglet weight at end of nursery period (Med ^b^; kg)	27.1	30.7
Piglet weight before slaughter (Ctl; kg)	140.3	125.6
Piglet weight before slaughter (Med; kg)	142.4	124.9
Vaccination age during nursery period (d)	35	32
Age at vitamins and selenium suppl. for 4 d (d)	115	NA ^c^
Age at salicylic acid treatment for 7 d/cough (d)	122	NA
Age at 2.5 ppm iodine treatment for 7 d/cough (d)	157	NA
Age at arrival at the nursery barn (d)	21	21
Age at beginning of antibiotic treatment (d)	40	36
Age at beginning of fattening phase (d)	71	69
Number of pens in nursery barn	34	40
Number of piglets/pen in nursery barn (0.29 m^2^/piglet)	15	15
Number of pens in finishing barn per experimental group	20	14
Number of pigs/pen in finishing barn	25	21
Number of pens sampled	10	10
Feed withdrawal time (h) before transport:		
Nursery to the finishing barn	12	12
Finishing barn to the abattoir	5	6
Total feed withdrawal time (h) before slaughter	14	16
Distance from the farrowing to the nursery barn (km)	7.6	98
Distance from the nursery to the finishing barn (km)	10	50
Distance from the finishing barn to the abattoir (km)	12	40
Number of animals sent to the abattoir (Ctl)	198	165
Number of animals sent to the abattoir (Med)	157	160

^a^ Animals came from two commercial farrowing barns: one with an average health status (AS) and one with a lower health status (LS). These farms were selected from 126 farms by experienced veterinarians based on their health status history. ^b^ One experimental group from each farrowing barn received, ad libitum in their feed, a prophylactic administration of 660 g of chlortetracycline calcium complex per tonne of feed (Med) at weaning for a period of 21 days. The control group (Ctl) from each farrowing barn did not receive the chlortetracycline treatment. ^c^ NA = not applied.

## Data Availability

The data presented in this study are openly available in NCBI Sequence Read Archive database at https://www.ncbi.nlm.nih.gov/sra (accessed on 1 September 2024), reference number PRJNA1142024.

## Supplementary Materials

The following supporting information can be downloaded at: https://www.mdpi.com/article/10.3390/antibiotics13100997/s1, Figure S1: Boxplots of the alpha diversity of the microbial community of the samples grouped by treatment and farrowing barn of origin. On the y axis can be found the (A) Shannon diversity index or the carcass samples, (B) Shannon diversity index for the loin samples, (C) Simpson diversity index for the carcass samples, (D) Simpson diversity index for the loin samples. The median is indicated by a bar in the box. The box extends from the 1st to the 3rd quartiles, while the whiskers extend to the maximum and minimum values.; Table~S1:~Feeding phases served throughout the growth of pigs from weaning to slaughter for pigs originating from both the average sanitary status farrowing barn (AS) and the lower sanitary status farrowing barn (LS).; Table S2: Number of sequencing reads retained at different stages of bioinformatics processing.
