# Supplementary material for: Resilience of Loin Meat Microbiota and of Resistance Genes to a Chlortetracycline Treatment in Weaned Piglets
Source: Antibiotics (Basel). 2024 Oct 21;13(10):997. doi: 10.3390/antibiotics13100997 (PMC11504350; doi:10.3390/antibiotics13100997)
Supplement: Supplementary file 1 [file antibiotics-13-00997-s001.zip › Supplementary_files/Supplementary_Table_1.pdf]

Table S1. Feeding phases served throughout the growth of pigs from weaning to slaughter for pigs originating from both the average sanitary status farrowing barn (AS) and the lower sanitary status farrowing barn (LS)

Housing	Feed commercial name	Age (day; LS)	Age (day; AS)	Selenium (mg/kg)
Nursery	Poupon plus feed	21-28	21-27	0.3
	ACCEL piglet feed	29-34	28-40	0.5
	Piglet feed phase 2 plus [1]	35-55	41-62	0.3
	Piglet feed phase 2 plus	56-67	63-69	0.3
Finishing	Hog feed phase 3	68-80	70-90	0.3
	Hog feed phase 4	81-104	91-116	0.3
	Hog feed phase 5	105-119	117-134	0.5
	Hog feed phase 6	120-147	135-157	0.5
	Hog feed phase 7	148-151/158	158-174	0.5

[1] A supplement of chlortetracycline (660 g per ton of feed) was added in the feed of the Med group.
