# Supplementary material for: Resilience of Loin Meat Microbiota and of Resistance Genes to a Chlortetracycline Treatment in Weaned Piglets
Source: Antibiotics (Basel). 2024 Oct 21;13(10):997. doi: 10.3390/antibiotics13100997 (PMC11504350; doi:10.3390/antibiotics13100997)
Supplement: Supplementary file 1 [file antibiotics-13-00997-s001.zip › Supplementary_files/Supplementary_Figure_1.pdf]

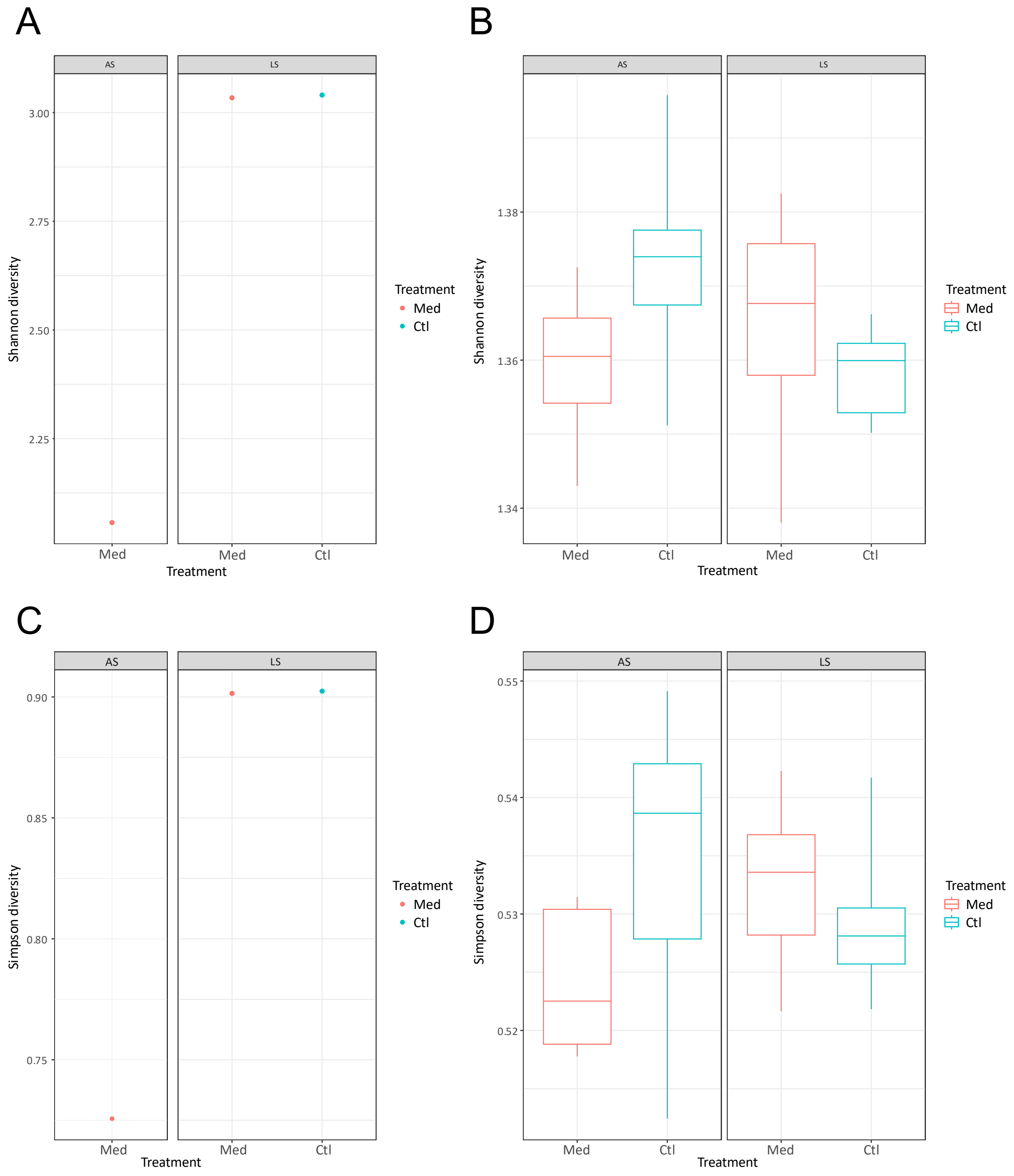

Figure S1. Boxplots of the alpha diversity of the microbial community of the samples grouped by treatment and farrowing barn of origin. On the y axis can be found the (A) Shannon diversity index for the carcass samples, (B) Shannon diversity index for the loin samples, (C) Simpson diversity index for the carcass samples, (D) Simpson diversity index for the loin samples. The median is indicated by a bar in the box. The box extends from the 1st to the 3rd quartiles, while the whiskers extend to the maximum and minimum values.
